# Adherence to medication in the community: audit cycle of interventions to improve the assessment of adherence

**DOI:** 10.1192/pb.bp.115.053520

**Published:** 2017-02

**Authors:** Saeed Farooq, Abid Choudry

**Affiliations:** 1 Keele University; 2 Leicestershire Partnership NHS Trust

## Abstract

**Aims and method** To investigate whether medication adherence is monitored during follow-up in out-patient reviews. A retrospective audit was carried out with a sample of 50 follow-up patients with a diagnosis of schizophrenia or schizoaffective disorder. Following this, interventions were made prior to the re-audit (including text messaging clinicians and prompt sheets in the out-patient department to encourage adherence discussions).

**Results** There was an improvement on all the standards set for this audit following the interventions. More doctors had discussed medication adherence (62% second cycle *v.* 50% first cycle) with their patient and there was increased discussion and documentation regarding medication side-effects (60% second cycle *v.* 30% first cycle). More clinicians discussed the response to medication (60% second cycle *v.* 46% first cycle).

**Clinical implications** Treatment adherence is not regularly monitored or recorded in clinical notes in routine psychiatric out-patient appointments. This highlights the need for regular training to improve practice.

The World Health Organization defines adherence as the extent to which a person's behaviour – taking medication, following a diet, and/or executing lifestyle changes – corresponds with agreed recommendations from a healthcare provider.^1^ Although often used interchangeably with the term ‘compliance’, adherence is preferred by many as it acknowledges the patient's role in the decision-making process.^2^ It has been claimed that increasing the effectiveness of adherence interventions may have a far greater impact on the health of the population than any improvement in specific medical treatments.^3^

Non-adherence to treatment is a major barrier to effective treatment in psychiatry, particularly in individuals with psychotic disorders. Rates of non-adherence vary between 24 and 40% based on medication refill rates available from pharmacy records.^4,5^ This is understandable in view of the different populations, variety of diagnoses, variable follow-up periods and, most importantly, the different definitions and measurement methods used in research.^6^ It has been reported in the literature that non-adherence rates to medication for bipolar disorder typically range between 20 and 60%, with an average of 40%.^7–9^ In schizophrenia, a systematic review of 39 studies reported a mean rate of medication non-adherence at 41%.^10^ When the analysis was restricted to the five methodologically most rigorous studies, which included defining adherence as taking medication at least 75% of the time, the non-adherence rate increased to 50%.^10^ Non-adherence to treatment in schizophrenia is often associated with potentially severe clinical consequences.^11,12^ It is also estimated that up to 40% of the total annual cost of schizophrenia, which amounts to £400 million in the UK, could be due to non-adherence to treatment.^13^

The National Institute for Health and Care Excellence (NICE) guidelines^14,15^ recommend that treatment adherence be regularly monitored in patients with schizophrenia but there is relatively little information about how this is done in clinical practice. In general, doctors uniformly underestimate the degree of non-adherence in their patients.^16^

## Aims

This audit was aimed at investigating whether medication adherence is monitored during the follow-up of patients diagnosed with schizophrenia or schizoaffective disorder. We also evaluated the ways in which medication adherence is discussed during out-patient reviews and recommend practice improvement.

## Method

We conducted a retrospective audit in 50 patients presenting to the out-patient follow-up clinic in two UK community mental health teams providing treatment for patients within the complex care team. The clinics were based in the Black Country Partnership NHS Foundation Trust. Only patients with a diagnosis of schizophrenia or schizoaffective disorder who were currently under out-patient follow-up were included. A random sample of patients was generated by taking every fifth patient from the list provided by the clinic administrator. The last clinic letter was reviewed to collect data via the medical notes and electronic healthcare records.

The standards were identified using the NICE guidelines on medicines adherence and treatment of psychosis and schizophrenia.^14,15^ The guidelines have emphasised what should be reviewed and discussed in out-patient clinics. Specifically, they recognise that the treatment should be regularly and systematically reviewed to monitor treatment adherence. During the titration of treatment the following should be regularly monitored:

- response to treatment, including changes in symptoms and behaviour
- side-effects of treatment
- emergence of movement disorders
- weight
- waist circumference
- pulse and blood pressure
- adherence
- overall physical health.

The NICE guideline on treatment adherence explicitly states that when reviewing medication the clinician should enquire about adherence:

‘If non adherence is identified, clarify possible causes and agree any action with the patient. Any plan should include a date for a follow up review.’^15^

As a consequence, the standards set out for this audit were that:

- 100% of patients should have a discussion with the doctor regarding the medication, including response and side-effects
- 100% of patients should have a discussion with the doctor regarding adherence to medication
- if medication is stopped, reasons for this should be explored.

In light of this we constructed a data collection tool for the audit (Box 1).

**Box 1** Information gathering tool for the audit

- Was adherence to medication discussed?
- Did the clinician ask the patient about any periods when they had missed taking medication?
- Did the patient mention missing any medication and was this discussed with the patient?
- Had the patient missed any medication over the past month?
- Were side-effects of the medication discussed?
- Did the clinician ask the patient whether these side-effects had impact on their adherence?
- If non-adherence was noted to be a significant problem, were any strategies to improve adherence discussed?
- Was the response to medication discussed?
- If medication was stopped, were reasons for this explored?

## Results

### Audit cycle 1

Data were collected over a period of 4 weeks. The results showed that adherence was discussed and documented only in 50% of consultations, side-effects were discussed only in 30% of consultations and response to medication was discussed in 46% of cases. Further questioning in terms of assessing adherence appeared to be poorly done, with less than 10% of consultations assessing adherence in greater detail.

The results of this initial audit highlighted either a gap in clinical practice or poor documentation. The results were discussed with colleagues in the weekly audit meeting in the hospital and recommendations were made to improve the practice (Box 2)

**Box 2** Recommendations following the first audit cycle

1. Provide formal training to doctors regarding assessing adherence during the induction for junior trainees and through a session delivered in the local teaching programme for the rest of the clinicians.
2. Provide a list of questions to be asked regarding adherence in clinics.
3. Provide a text reminder to doctors on clinic days to remind them to assess adherence.
4. Re-audit following implementation of changes.

As a consequence of the initial audit, a change in practice was brought about by introducing information sheets in each clinic room with possible questions concerning adherence to ask when assessing patients in clinic. A brief session on adherence was also added to the junior doctor induction. Then, a text message reminder was sent to colleagues. This included consultants, specialty doctors and trainees (core and foundation year 2) at the start of each clinic for a period of 3 months. The text message was very brief, reminding colleagues to discuss adherence with their patients in clinic. The colleagues' consent was sought prior to this 3-month trial. We initially planned to send the text reminders using NHS.net, which provided such a service, but that stopped in early 2015. As a result, we sent out a group message using the work mobile phone.

Following this period a re-audit was carried out. It again focused on patients with schizophrenia and schizoaffective disorder but only spanned the intervention period of the prior 3 months.

### Re-audit

**Fig. 1:**
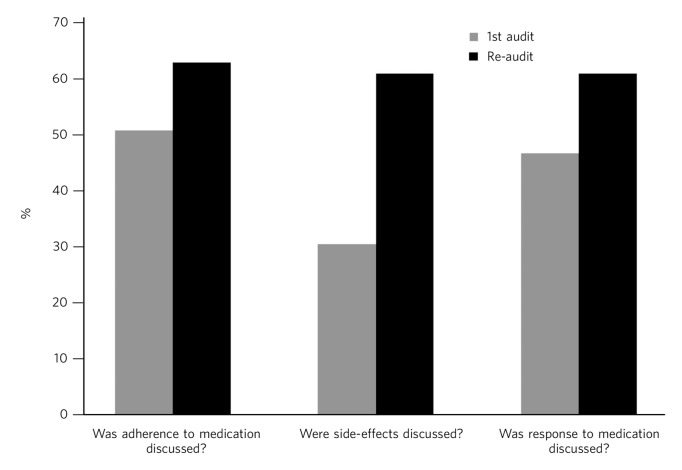
Comparison of key audit results.

As before, a random sample of 50 patients was selected. A similar procedure was carried out, but only patients reviewed after the initial audit were included. The same data collection tool was used (Box 1). The results are illustrated in Fig. 1.

The results indicated an improvement in all three key comparisons. More doctors had discussed medication adherence with their patient over the past 3 months (62% *v.* 50%, *P* = 0.22), and there was increased discussion with patients and documentation of side-effects (60% *v.* 30%, *P* = 0.0025). More clinicians discussed the response to medication with their patients in the second audit (60% (*n* = 30) *v.* 46% (*n* = 23), *P* = 0.16). The results regarding further questions about adherence continued to be poor, with only 4% (*n* = 2) asking about whether the patient had missed any doses of medication. However, it was felt this may be secondary to a lack of documentation rather than being a true reflection of practice.

*P*-values revealed a significant difference in the two audits for the discussion about side-effects, with an improvement noted following the interventions. However, there was no significant difference on discussions about adherence and response to medication between the two audits.

## Discussion

Improving treatment adherence is at the heart of clinical psychiatry. It requires building a therapeutic relationship with patients, understanding their needs and tailoring treatment accordingly. Monitoring treatment adherence is a continuous process during which the need to continue medication in the long term is regularly stressed. We need to identify the barriers and help patients and carers to overcome these. This can only be achieved if the treatment is regularly monitored for its efficacy, side-effect burden and acceptability to patients.

This audit presents a rather disappointing picture of the conversation about treatment adherence, which is not common in clinical encounters. Such discussions took place in just half of the consultations, whereas questions about possible side-effects and response to medication were raised even less frequently. The first variable improved to about 60% after regular reminders and inclusion of this topic in the junior doctor induction, but this result was not statistically significant. However, the results were statistically significant concerning discussions about side-effects, with a significant improvement noted following the interventions. This perhaps highlights the need for regular training to improve the monitoring of treatment adherence.

It has been shown that simple questions about different aspects of medication-taking behaviours can be effective in improving treatment adherence.^17^ Most of the information regarding assessment of adherence is based on clinical experience or limited research. Clinicians can start by asking patients ‘Have you missed any pills in the past week?’ A positive response indicates there may be a problem with adherence. Clinicians should bear in mind that patients tend to overestimate their actual adherence to therapy and that the accuracy of the self-report depends on the patient's cognitive abilities, attitudes and openness towards the therapist.^6^ Some simple questions that can be asked in routine clinical care include: ‘How are you taking your medications?’, ‘Have you ever forgotten to take your medications?’, ‘Are you experiencing any adverse drug reactions?’, ‘How are you feeling since you started the medication?’^6^

The results of the audit should be interpreted in the light of some limitations. It is possible that these questions are asked but not regularly documented. Patient medication adherence was not directly measured in the audit. The study had a small sample size that represents a snapshot of clinical encounters, which may not be generalisable. The discussion about treatment adherence may have taken place in different settings such as the in-patient setting or with other members of the multidisciplinary team such as the community psychiatric nurse. We used text message reminders, which has helped the clinicians ask about adherence. Text messages have often been used to remind patients abut medication, but they have not been commonly used to alter the clinicians' behaviour, and can prove a simple and effective method for improving adherence with good practice.
